# Expression changes of Tim-3 as one of supplementary indicators for monitoring prognosis of liver pathological changes in chronic HBV infection

**DOI:** 10.1186/s12879-022-07841-1

**Published:** 2022-11-11

**Authors:** Shanshan Wu, Xinfang Du, Guohua Lou, Shuihong Yu, Kecong Lai, Jinjin Qi, Shujun Ni, Zhi Chen, Feng Chen

**Affiliations:** 1 State Key Laboratory for Diagnosis and Treatment of Infectious Diseases, National Clinical Research Center for Infectious Diseases, Collaborative Innovation Center for Diagnosis and Treatment of Infectious Diseases, The First Affiliated Hospital, College of Medicine, Zhejiang University, Hangzhou, 310003 China; 2 Department of Infectious Disease, Beilun People’s Hospital, Ningbo, 315800 China; 3 Department of Gastroenterology, Beilun Second People’s Hospital, Ningbo, 315809 China

**Keywords:** Chronic hepatitis B, T-cell immunoglobulin and mucin domain containing molecule 3, Liver biopsy, Fibrosis, Follow-up

## Abstract

**Purpose:**

This study was designed to analyze the liver tissue changes among the CHB patients who received treatment for at least 6 months and follow-up for at least 1 year, together with the correlation between the different disease condition and serum markers.

**Methods:**

One-hundred and eighty-five CHB patients underwent antiviral therapy for at least 6 months were enrolled. In the 12-month follow-up, ultrasonography-guided biopsy was performed. The patients were grouped based on the serum markers and pathological changes in liver tissues. Then we determined the serum markers, virological tests and Tim-3 expression among these groups.

**Results:**

Antiviral therapy significantly reduced liver inflammation indicators and serum Tim-3 level. However, the fibrosis process of liver tissue was not changed, and there are still disputes on the serum marker and hepatic lesion outcomes. Under normal liver function or negative hepatitis B e antigen (HBeAg) of CHB patients, there might be consensus between Tim-3 change and liver pathological outcome. According to the liver tissue inflammation and fibrosis conditions, Tim-3 was positively correlated with liver function indices. Besides, it was also related to fibrosis stage and inflammation grade.

**Conclusion:**

There were inconsistent changes between serum markers and liver tissue conditions after anti-viral therapy. Tim-3 expression was more suitable to indicate the changes of liver inflammatory and fibrosis response to some extent than ALT and AST. It may serve as a certain indicator to predict the CHB prognosis, which could be used as one of the monitoring indicators in liver pathological changes of chronic HBV infection, especially in monitoring liver tissue inflammation.

## Introduction

Immune system is closely participated in the regulation of inflammatory reactions during the infection of hepatitis B virus (HBV). HBV carriers can endure for several years without clinical manifestations [1]. Not all patients infected with HBV develop serious complications, but a part of them (15–40%) have no effective immune responses against virus. This would lead to potential adverse sequelae such as liver failure (LF), liver cirrhosis (LC) and hepatocellular carcinoma (HCC) [2–4]. In China mainland, HBV infection is responsible for about 60% of LC and 80% of HCC [5]. Thus, fibrosis evaluation and timely intervention are necessary for the patients with chronic hepatitis B (CHB). To date, liver biopsy is the gold standard for evaluating the progression of liver diseases. However, its clinical application is hampered due to its traumatic features and poor repetition within a short time period. As is known to all, serological indicators for HBV infection such as aspartate aminotransferase (AST) and alanine aminotransferase (ALT) were in the normal ranges in patients with mild infection, however, this does not mean that the disease is completely cured. Besides, partial patients showed deterioration and progression of inflammation in liver tissues, and the sensitivity of ALT level was not high. On this basis, extensive attempts have been made to find new serum biomarkers to evaluate the progression of hepatic diseases.

T-cell immunoglobulin and mucin domain containing molecule 3 (Tim-3), a member of the Tim family, is newly identified surface marker expressing on type I helper T lymphocytes (Th1) [6]. The common structure of Tim-3 is consisted of immunoglobulin variable domain (Ig V), mucin stalk, transmembrane region and cytoplasmic tail [7]. Tim-3 lacking the mucin and transmembrane region is found in a soluble form [8]. Soluble Tim-3 (sTim-3) can reduce IL-2 production by T cells under in vitro conditions [9]. Tim-3 ligands could mediate signal transduction via interacting with the Ig V domain of Tim-3. In the past decades, the immunomodulatory effects of Tim-3 on HBV infection have become a hotspot. Studies have suggested that Tim-3 serves as a negative regulator can induce the depletion of Th1 cells upon binding to its ligand galectin-9 (Gal-9) [10]. Tim-3/Gal-9 signaling pathway induces T cell senescence [10] and plays as alternative checkpoint in antitumor immunity and cancer immunotherapy [11]. The downstream signaling pathways promoted by Tim-3 in both adaptive and innate immune cells remain to be clarified. The immune dysfunction induced by HBV infection may be related to the high protein levels involved in the Tim-3/Gal-9 signaling pathway [12]. The dysfunction of CD8+ T-cells in patients infected with chronic HBV could be reversed by blocking Tim-3 -mediated signaling pathway [12–14]. Tim-3 expression is positively associated with the level of ALT in CHB patients [15]. Indeed, Tim-3 acts as a checkpoint receptor in human clinical trials [7]. It has been reported that sTim-3 served as the dominant plasma soluble- checkpoint receptor (CR) in alcohol-related liver disease (ALD) [16]. Soluble-CRs were strongly correlated with pro-inflammatory cytokines and disease severity. It was reported that remarkable elevation of Tim-3 level correlated with elevated risks of hepatocellular carcinoma among CHB patients [17]. Tim-3 polymorphisms may affect disease susceptibility and HCC traits associated with HBV infection [18]. All these demonstrated that Tim-3 might affect the susceptibility of chronic HBV infection and indicated its role in disease progression. Up to now, little is known about the relationship between serum sTim-3 expression and liver pathology in CHB patients. The roles of sTim-3 are still not well defined in hepatic fibrosis and inflammation of CHB patients who received anti-viral therapy and presented various pathological changes in liver tissues. In this study, we investigated the potential relationship between serum Tim-3 and other indicators, as well as the role of Tim-3 in monitoring of CHB.

## Materials and methods

### Subjects

One hundred and eight-five CHB patients (male: 105; female: 80; age: 41.52 ± 11.92 years) presented to the Outpatient Department of our Hospital from May 2011 to December 2014 were included in this study. CHB diagnosis was given based on the guidelines of the American Association for the Study of Liver Diseases (AASLD) [19].

Patients met the following inclusion criteria were included in this study: positive hepatitis B surface antigen (HBsAg) for more than a half year; antiviral agents administration before enrolling into this study, such as nucleotide analogs; free of LC and HCC at presentation; no other chronic liver disorders such as drug-induced hepatitis, autoimmune liver disease or alcoholic hepatitis, or liver injuries by other causes; no other hepatitis virus infection (e.g. hepatitis A, C, D and E virus), or co-infection with human immunodeficiency virus such as HIV infection.

### Laboratory investigations

The concentration of albumin (ALB), total protein (TP), globulin (GLB), ALT, AST, direct bilirubin (DBIL), indirect bilirubin (IDBIL), total bilirubin (TBIL), total bile acids (TBA), glutamyl transpeptidase (γ-GT), alkaline phosphatase (ALP), cholinesterase (ChE) and glycyl-proline-dipeptidyl aminopeptidase (GPDA) was measured to evaluate the liver function using the Roche automatic biochemical analyzer (Roche, CA, USA). Quantification of HBsAg, hepatitis B e antigen (HBeAg), antibodies against hepatitis B surface antigen (anti-HBs), antibodies against hepatitis B core antigen (anti-HBc) and antibodies against hepatitis B e antigen (anti-HBe) was carried out using chemiluminescent enzyme immunoassay (CLEIA, Abbott Laboratories, Tokyo, Japan). CLEIA was utilized to determine the concentration of serum alpha-fetoprotein (AFP). HBV-DNA level was determined by one-step method of Real-Time PCR on an ABI-7500 system. All operations were strictly in accordance with the standardized operating procedures (SOP).

### Pathological analysis

Percutaneous ultrasound-guided puncture was performed to obtain a small piece of liver tissue. The specimens for liver biopsy were conventionally stained with hematoxylin and eosin. Five observational fields were randomly selected under the microscope (× 400, Olympus BX51, Tokyo, Japan). The degree of fibrosis and inflammatory reactions in the hepatic tissues of CHB patients were measured using the guidelines of CHB prevention and treatment updated in 2015 [20]. The pathological results were judged by two pathologists blinded to this study. Fibrotic stage and inflammation grade included 0, 1, 2, 3, 4 stage (i.e. S1–4 or G1–4). Based on the Scheuer scoring system, S0 represented no fibrosis and G0 represented no hepatic necroinflammation. G0 and G1 were mild inflammatory reactions, in which patients were classified as mild inflammation group. G1–2, G2 and G2–3 were defined as moderate inflammatory response and G3 was defined as severe inflammatory reaction, in both of which patients were classified as moderate to severe inflammation group. Based on the degree of fibrosis, patients of S0, S0–1 and S1 degree were classified as no significant fibrosis group. Patients of S1–2 and above degree were classified as significant fibrosis group.

### Definition of histological healing

According to the pathological inflammation or fibrotic grade comparison between baseline level and 12-month follow-up level, patients were categorized into three groups: Stable condition which was defined as stable condition in pathological inflammation or fibrotic grade. Clinical remission which was defined as a remission in the inflammation or fibrotic grade. Clinical disease progression which was considered as deterioration in inflammation or fibrotic grade.

### Determination of Tim-3 expression in patient serum

Serum Tim-3 in CHB patients was measured using the commercial Tim-3 ELISA kit (catalog No. ELH-TIM3, RayBio, USA) at the beginning of the study and at the end of the 12-month follow-up, respectively. In brief, sample diluent was used to dilute the serum samples. A microtiter plate reader (Bio-Rad, Hercules, CA) was utilized to measure the absorbance at 450 nm. Serum samples were triplicated in the experiment and the mean absorbance was calculated. Tim-3 level was quantified with a calibration curve. Then the standard curve was plotted on log–log graph paper using Curve Expert 1.3 software.

### Statistical analysis

SPSS 21.0 software was utilized for the statistical analysis. All patients were classified based on pathological analysis, statistical analysis of basic clinical information, such as liver function, HBV-DNA, and AFP. The differences of serum Tim-3 expression between patients with various liver fibrosis and different inflammatory reactions in the liver tissues were compared and analyzed. The correlation between serum Tim-3 expression and liver function indices (e.g. HBV-DNA and AFP) was analyzed by Spearman correlation analysis. Student’s t-test or Chi square test was utilized for the inter-group comparison. The rank distribution data was analyzed with the Fisher exact probability test or Mann–Whitney U test for the unpaired samples. For the comparison of paired samples, Wilcoxon signed rank test was used. *P* < 0.05 was considered to be statistically significant.

## Results

### Demographic and clinical characteristics

Finally, 185 CHB patients underwent antiviral treatment for at least 6 months were included. Table 1 summarized the patient characteristics before treatment and at month 12 after treatment. Subjects were sex-matched with a female/male ratio of 80 (43.24%)/105 (56.76%). Their average age in the male and female were (42.61 ± 10.56) years. and (40.70 ± 12.84) years. No statistical differences were noticed in the gender and age between the patients (*P* > 0.05). Serological results demonstrated that, compared with the baseline, the levels of hepatitis-related inflammatory indicators all showed decrease in the 12-month follow-up. There were significant differences in the ALT, AST, γ-GT, Tim-3 (*P* < 0.01), AFP (*P* < 0.01), HBV-DNA (*P* < 0.01) and inflammation grading (*P* < 0.01) of liver pathology. Antiviral therapy could effectively enhance the inflammatory response of hepatitis B patients and could delay the progression of the hepatitis disease.

**Table 1:** Comparison of clinical information and serum liver function indices of 185 patients with CHB

Index	Baseline level (95% CI)	12-Months follow-up (95%CI)
TP, g/L	77.83 ± 5.97 (76.94–78.69)	77.82 ± 7.56 (76.70–78.92)
ALB, g/L	49.10 ± 4.38 (48.46–49.74)	52.46 ± 32.27 (47.76–57.23)
GLB, g/L	28.73 ± 4.48 (28.06–29.36)	27.71 ± 4.21*(27.07–28.31)
A/G	1.75 ± 0.31 (1.71–1.80)	1.93 ± 1.36**(1.74–2.14)
ALT, U/L	50.75 ± 76.07 (39.83–62.13)	25.32 ± 25.32**(21.69–29.11)
AST, U/L	47.53 ± 65.94 (38.00–57.34)	26.25 ± 14.62**(24.18–28.46)
ALP, U/L	86.44 ± 32.42 (82.08–91.53)	80.85 ± 27.03(77.23–85.11)
ChE, U/L	8540.39 ± 2189.15 (8221.87–8863.37)	8898.41 ± 2102.60 (8584.14–9199.52)
TBA, μmol/L	20.49 ± 30.77 (16.09–25.11)	15.71 ± 21.99*(12.61–19.05)
TBIL, μmol/L	9.05 ± 8.12 (7.85–10.23)	8.45 ± 4.37(7.84–9.12)
DBIL, μmol/L	4.12 ± 5.82 (3.28–4.99)	3.50 ± 1.87(3.25–3.79)
IDBIL, μmol/L	4.93 ± 4.37 (4.26–5.54)	4.95 ± 3.42(4.46–5.46)
γ-GT, U/L	41.52 ± 41.73 (35.60–47.82)	27.11 ± 25.18**(23.49–30.86)
GPDA, U/L	100.67 ± 27.49 (96.78–104.81)	93.81 ± 21.52*(90.84–97.09)
AFP, ng/mL	7.63 ± 16.87 (5.20–10.13)	3.93 ± 7.94**(2.78–5.11)
HBsAg, IU/mL	14,194.86 ± 24,143.84 (10,711.40–17,771.29)	7521.35 ± 16,629.74**(5107.19–9982.06)
anti-HBs, mIU/mL	1.41 ± 9.62 (0.02–2.84)	0.90 ± 4.22(0.29–1.53)
anti-HBc, S/CO	8.54 ± 1.25 (8.35–8.71)	8.25 ± 1.25**(8.06–8.43)
HBeAg, PEIU/mL	304.51 ± 547.03 (227.74–387.92)	151.52 ± 412.97*(92.54–213.59)
anti-HBe, S/CO	10.39 ± 17.32 (7.90–12.98)	5.26 ± 12.46(3.49–7.14)
HBV-DNA, IU/mL	2.91E8 ± 8.31E8 (1.68E8-4.11E8)	1.62E7 ± 8.25E7**(4.25E6-2.85E7)
Tim3, pg/mL	879.23 ± 646.15 (787.90–977.09)	655.98 ± 399.33**(600.65–717.37)
Inflammation grading	1.61 ± 0.75 (1.50–1.72)	1.38 ± 0.55**(1.30–1.46)
Fibrosis stage	1.31 ± 1.07 (1.16–1.47)	1.34 ± 0.95 (1.20–1.48)

*CI* confidence interval

*P < 0.05 versus the baseline level; **P < 0.01, versus the baseline level

### Changes of inflammation and fibrosis in liver tissues

The liver biopsy results of 185 patients showed that for the inflammation grade, 5 showed G0, 3 cases of G0–1, 83 cases of G1, 19 cases of G1–2, 52 cases of G2, as well as 7 cases of G2–3 and 16 cases of G3 in baseline level while 116 cases of G0 and G1, 94 cases of G2 and G3 after 12-month follow up. For the baseline fibrosis, 43 cases were of S0 degree, 19 cases of S0- 1, 55 cases of S1, 24 cases of S1–2, 18 cases of S2, 9 cases of S2–3, 10 cases of S3, 5 cases of S3–4, and 2 cases of S4, respectively. After the 12-month follow-up, 105 cases were of S0 and S1 degree and 80 cases were of S2 degree or more (Table 2). Liver inflammation in CHB patients showed significant improvement after antiviral therapy. However, the fibrosis process of liver tissue was not changed.

**Table 2:** Cases of different pathological changes of liver tissue

Pathological outcome	Grading group	Cases of baseline level	Cases after 12-month follow-up
Inflammation	Mild inflammatory response	91	116
	Moderate to severe inflammatory response	94	69
Fibrosis	No significant fibrosis	117	105
	Significant fibrosis	68	80

### Comparison of hepatitis-related inflammatory indices

**Fig. 1:**
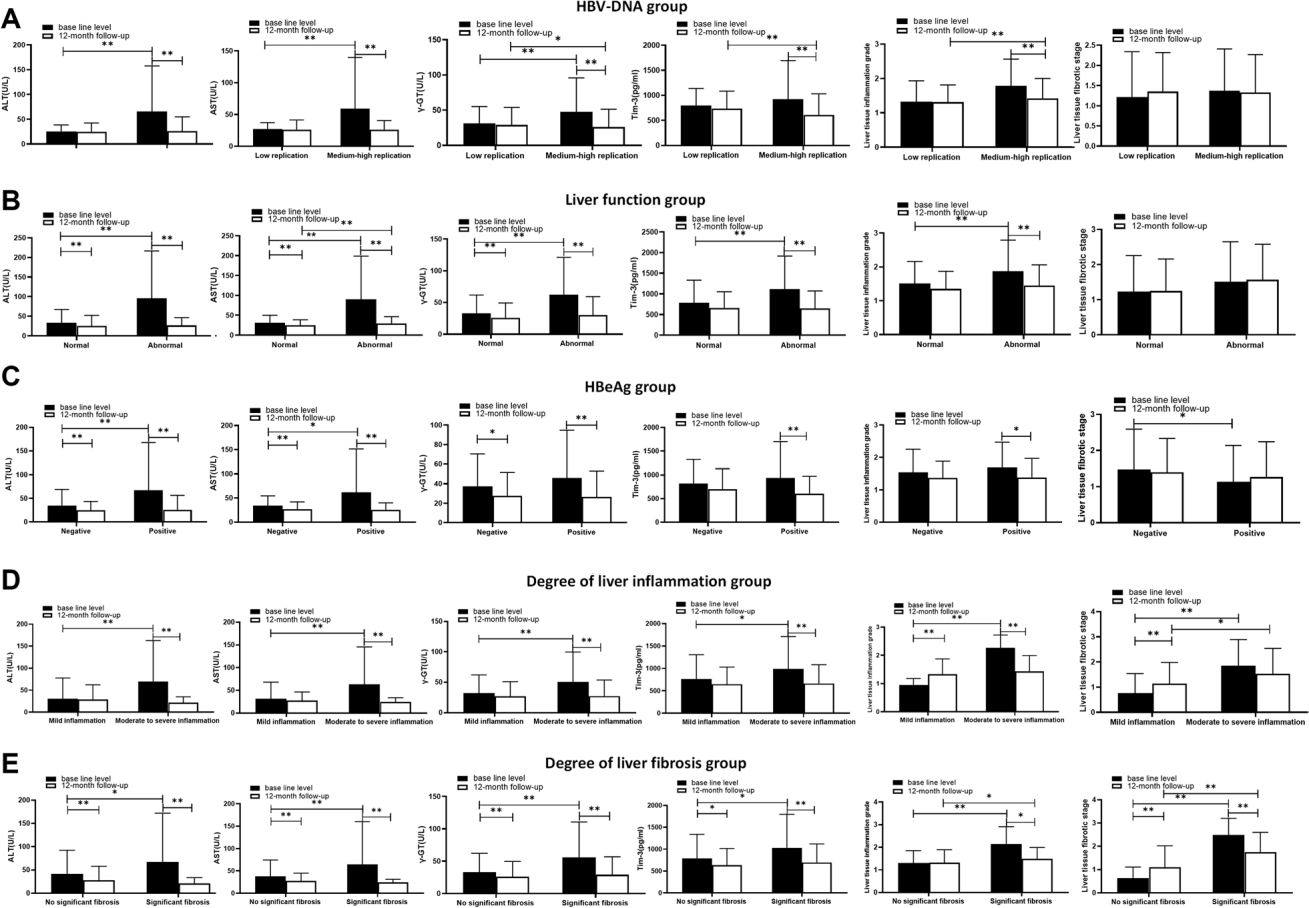
Comparison of testing indexes between different groups in patients with hepatitis B in different states. **A** The levels of testing indexes in patients with hepatitis B who were grouped according to HBV-DNA. Low replication (HBV-DNA ≤ 1000 IU/mL) and medium–high replication (HBV-DNA > 1000 IU/mL). **B** The levels of testing indexes in patients with hepatitis B who were grouped according to liver function. Normal (ALT and AST ≤ 40 U/L) and Abnormal (ALT or AST > 40 U/L). **C** The levels of testing indexes in patients with hepatitis B who were grouped according to HBeAg. Negative (< 0.18 PEIU/ml) and positive (≥ 0.18 PEIU/mL). **D** The levels of testing indexes in patients with hepatitis B who were grouped according to the degree of liver inflammation. Mild inflammation (≤ G1) and moderate to severe inflammation (> G1). **E** The levels of testing indexes in patients with hepatitis B who were grouped according to degree of liver fibrosis. No significant fibrosis (≤ S1) and significant fibrosis (> S1). Error bars denote the mean ± standard deviation; *P < 0.05, **P < 0.01

Based on HBV-DNA levels, patients were divided into low replication group and moderate-high replication group. Patients were classified into normal and abnormal groups by the liver function. Significant elevation was noticed in the levels of inflammatory indices in moderate-high HBV-DNA replication group, liver dysfunction group, HBeAg positive group, liver tissue moderate to severe inflammatory reaction groups and severe liver fibrosis group compared with the corresponding groups (Fig. 1). The expression of various inflammation indicators showed significant decline after continuous antiviral therapy. Meanwhile, the degree of liver tissue inflammation and fibrosis also showed improvement accordingly, but not all of them. We found that there was significant decline in the expression of ALT, AST, γ-GT in HBeAg negative group and normal liver function group compared with the baseline level (*P* < 0.05). After about 12 months treatment, the Tim-3 expression showed slight decline, while the inflammatory grade in the liver tissues showed improvement and the fiber classification showed increase. However, there were no statistical differences between liver tissue inflammation and liver tissue fibrosis when comparing with the baseline (*P* > 0.05). At the baseline level, there was significant decline in the expression of ALT and AST in HBeAg-negative group than that of HBeAg-positive group (*P* < 0.05 or *P* < 0.01), however, there were no significant differences between Tim-3 and liver tissue inflammation levels (*P* > 0.05). In other case groups, similar trends were noticed in the serum Tim-3 and other inflammatory indicators.

According to the histological healing, patients were categorized to three groups: stable condition, clinical remission and clinical disease progression condition (Table 3). Compared with the baseline level, the ALT, AST, γ-GT and Tim-3 in the improved condition group showed similar trend in 12-month follow-up. After about 12 months treatment, the AST, γ-GT, ALT, and Tim-3 showed significant decline compared with the baseline (*P* < 0.01). In the stable and progressive conditions, there was similar trend in the Tim-3 expression and the pathological changes in hepatic tissues, except the ALT, AST and the pathological changes of the liver tissues (Table 3). These indicated that the Tim-3 was more suitable to demonstrate the pathological alternations of the liver tissues compared to the ALT and AST in the patients with specific CHB infection.

**Table 3:** Comparison of serum testing indexes expression changes in CHB patients with different pathological changes of liver tissue

Index	Stable condition(n = 46)		Clinical remission (n = 74)		Clinical disease progression (n = 65)	
	Baseline level (95%CI)	One year later (95%CI)	Baseline level (95%CI)	One year later (95%CI)	Baseline level (95%CI)	One year later (95%CI)
ALT, U/L	41.17 ± 65.88 (21.61–60.74)	25.26 ± 21.11^#^ (18.99–31.53)	66.55 ± 93.83 (44.82–88.29)	21.58 ± 13.66^##^ (18.42–24.75)	39.52 ± 55.32 (25.81–53.23)	29.61 ± 35.81 (20.24–38.49)
AST, U/L	39.09 ± 51.56 (23.77–54.40)	25.96 ± 18.01^##^ (20.61–31.30)	60.23 ± 83.49 (40.89–79.57)	23.96 ± 8.92^##^ (21.89–26.03)	39.05 ± 48.70 (26.98–51.11)	29.06 ± 16.79 (24.90–33.22)
γ-GT, U/L	33.72 ± 33.50 (23.77–43.67)	23.07 ± 14.57 (18.74–27.39)	50.62 ± 51.74 (38.63–62.61)	26.57 ± 27.40^##^ (20.22–32.92)	36.69 ± 31.62 (28.86–44.53)	30.58 ± 28.16 (23.61–37.56)
Tim3, pg/mL	800.93 ± 520.55 (646.34–955.51)	712.20 ± 428.81 (584.86–839.54)	991.41 ± 792.25 (807.86–1174.96)	644.67 ± 431.30^##^ (544.74–744.59)	806.94 ± 519.25 (678.28–935.60)	629.07 ± 337.39^#^ (545.47–712.67)
Inflammation Grade	1.30 ± 0.47 (1.17–1.44)	1.28 ± 0.46 (1.15–1.42)	2.15 ± 0.69 (1.99–2.31)	1.22 ± 0.48^##^ (1.11–1.33)	1.22 ± 0.60 (1.07–1.36)	1.63 ± 0.601^#^ (1.48–1.78)
Fibrosis Stage	1.24 ± 0.90 (0.97–1.51)	1.20 ± 0.86 (0.94–1.45)	1.97 ± 1.01 (1.74–2.21)	1.05 ± 0.98^##^ (0.83–1.28)	0.62 ± 0.74 (0.43–0.80)	1.77 ± 0.825^#^ (1.56–1.97)

^#^*P* < 0.05 versus the baseline level; ^##^*P* < 0.01 versus the baseline level

### Serum Tim-3 was associated with histological activity and may serve as a complement inflammatory marker for CHB

**Fig. 2:**
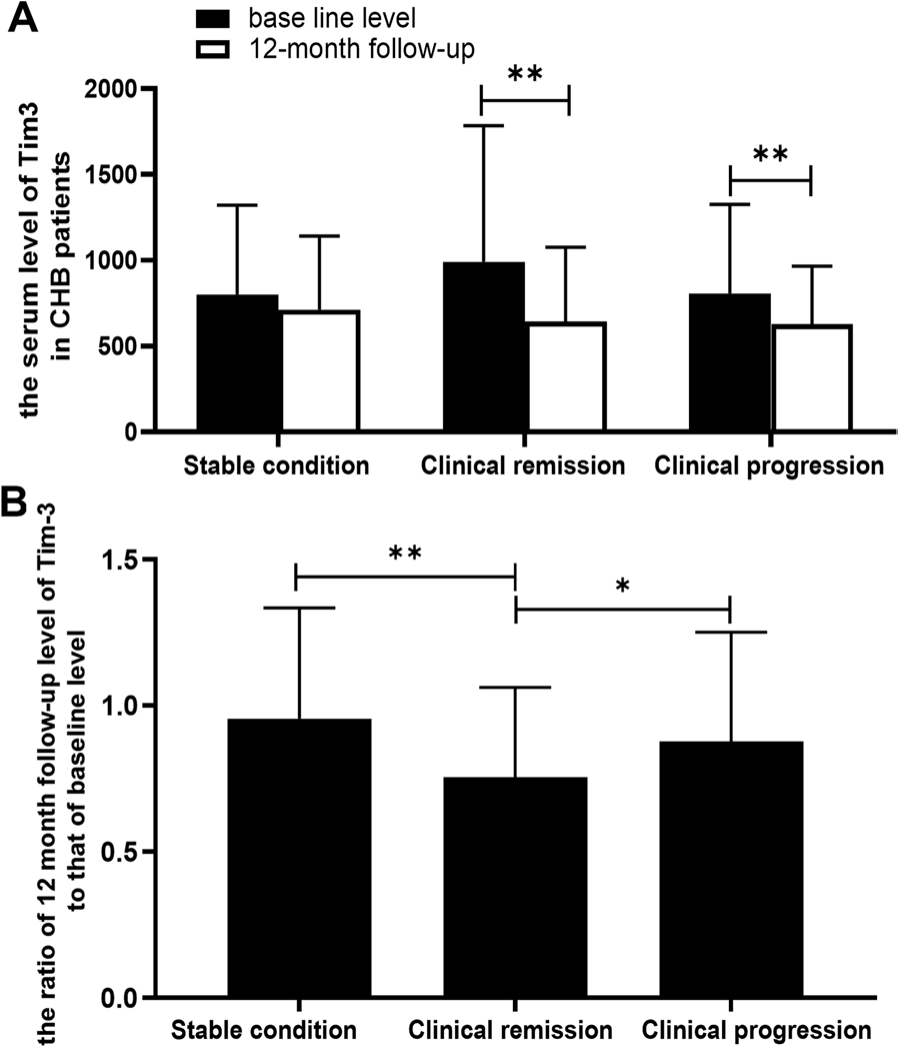
Tim-3 level in CHB patients with different prognosis condition after antiviral therapy before and after 12-month follow-up. **A** The levels of Tim-3 grouped according to pathological changes of liver tissues with stable condition, improved condition and progression condition. **B** Tim-3 ratio at 12-month follow-up level to that of baseline level in patients who were grouped according to pathological changes of liver tissue. *P < 0.05; **P < 0.01

Tim-3 level may increase with the severity of liver inflammation or liver fibrosis (Fig. 1D and E). Compared with the baseline level, the serological results indicated decrease of Tim-3 in the 12-month follow-up, and there were statistical differences in the moderate to severe inflammatory response group (*P* < 0.01, Fig. 1D). Tim-3 expression in moderate to severe inflammatory response group or significant fibrosis group was significantly higher compared with that in patients with mild inflammatory or fibrosis response at baseline (*P* < 0.05, Fig. 1D and E). On the contrary, compared with the baseline level, there was significant decline in the Tim-3 of the 12-month follow-up in patients of clinical remission group (*P* < 0.01) or clinical disease progression group (*P* < 0.01). No significant differences were noticed compared with the stable condition group (*P* > 0.05, Fig. 2A). Compared with the stable condition group, the ratio of Tim-3 at 12 month follow-up to that of baseline level showed decrease in clinical remission group and clinical disease progression group. Significant differences were noticed between the clinical remission group and stable condition (*P* < 0.01) or clinical disease progression group (*P* < 0.05, Fig. 2B). All these data implied that the Tim-3 levels showed significant increase in CHB patients. The reduction of Tim-3 may serve as a predictive factor for the prognosis of hepatitis B disease.

### Correlation analysis of serum Tim-3 and other test indices of CHB patients

A significant correlation was noticed between serum Tim-3 level and age (r = 0.27, *P* < 0.01). Besides, there was a positive correlation between liver function indices (i.e. ALB, GLB, ALT, AST, TB, DB, TBA, and γ-GT) and AFP (r = 0.299, *P* < 0.01) in the cases infected by chronic HBV. Moreover, there was a positive correlation between serum Tim-3 and inflammation grading (r = 0.22, *P* < 0.01) and fibrosis stage (r = 0.15, *P* < 0.05) in the CHB patients. No correlation was available between serum Tim-3 level and HBV-DNA load.

## Discussion

The severity and the outcome of the disease are still unclear even in the presence of clinical symptoms. In recent years, many studies have shown that the outcome of CHB is related to its clinical types, the immune status of the host and genetic factors, as well as the cellular immunity. The persistent existence of immune response is crucial for the progression of CHB to cirrhosis and liver cancer [21, 22]. To date, antiviral therapy has been considered as one of the most utilized treatment options for CHB, which can delay the disease progression in patients infected with hepatitis B. Therefore, it is necessary to monitor the disease condition in CHB patients. HBV-DNA is a direct indicator for HBV infection and replication [23]. Nowadays, ALT, AST and γ-GT are sensitive indicators for the evaluation of liver injury, however, they could not reflect the progression degree of liver pathology in an accurate manner. The inflammatory indicators of CHB patients enrolled in this study showed significant improvement after antiviral therapy, however, not all the changes were consistent with the pathological changes of hepatic tissues. Liver biopsy was performed to measure the extent of inflammation and fibrosis in hepatic tissues, which involved invasive procedures, together with a high cost and complicated tests. Therefore, it cannot be used as a routine screening tool in clinical practice. Other new markers are needed to evaluate the pathological alternation of hepatic tissues in clinical settings.

Tim-3 is structurally a member of the T-cell immunoglobulin mucin family localizing on chromosome 5q33.2. It is mainly expressed on the surface of Th l cells [6, 24], which exerts immunosuppressive roles in Th l cells by regulating the immune response negatively. Besides, it was expressed on a variety of non-specific immune response cells, such as natural killer cells [25], monocytes [6, 26], and melanoma cells [18, 27]. Tim-3 with a key role in regulating inflammation in terms of its non-canonical signaling. Polymorphism of Tim-3 gene involved in the determination of different functions [28, 29]. Tim-3 is highly expressed in liver tissues and tend to be markedly correlated with their gene polymorphisms in patients with HBV-induced HCC [30, 31]. It also has multiple different ligands, and would lead to different biological effects when combining with different ligands [8]. For instance, Gal-9 was the first ligand of Tim-3 and is enriched in cells that expressing in the liver [32]. Studies had reported that there were high affinity interaction between the Tim-3 Ig V domain and Gal-9, Tim-3/Gal-9 pathway mediated T cell exhaustion and induced apoptosis of Th1 cells [33]. Carcinoembryonic antigen cell adhesion molecule 1 (CEACAM1) is a ligand of Tim-3 that is necessary to mediate the suppressive function of T cells [34]. Binding of Tim-3 and CEACAM1 promotes temporal differences in Restimulation-induced cell death (RICD) sensitivity in the effector T cell response [35], which plays a key role in regulating anti-tumour immunity [34] and antiviral responses [36]. As another Tim-3 ligand, phosphatidylserine (PtdSer) may play a crucial role in regulating tolerance by clearing apoptotic cells [37, 38] and enhancing cross-antigen presentation [39]. High mobility group protein B1 (HMGB1) known to be a DNA binding protein was identified as the ligand of Tim-3. The results of structural and biological researches showed that the interaction of Tim-3 and HMGB1 contributed to maintain the stability of genome and regulate the transcription of nuclear [40, 41], but rare studies focused on their interaction. To date, little is known about how Tim3+ cells can discriminate between these ligands. The above evidences highlight the role of Tim-3-ligands interaction can potentially inhibit immune responses, which may have other functions in different immune-related diseases.

Numerous studies indicated that Tim-3 as an inhibitory receptor was closely associated with the pathogenesis of hepatic disease. The level of Tim-3 expressed by T cells in the patients with CHB is related to the severity of the disease [14].According to the previous description, Tim-3 closely involved in host immune regulation during HBV infection [12, 17]. Besides, the expression of Tim-3 in HCC tissues was significantly up-regulated compared with that of adjacent cancer and normal liver tissues [12]. Interferon-gamma (IFN-γ) secreted by tumor-infiltrating T cells stimulated Gal-9 expression on antigen presenting cells (APCs) [12]. There is high Gal-9 expression in Kupffer cells (KCs) of liver. Tim-3/Gal-9 signaling could induce T cell apoptosis in a co-localization pattern in HCC. Blockade of Tim-3 pathway can ameliorate inflammatory cytokine IFN-γ production [42] and indirectly affect HBV neutralizing antibody production. Moreover, Tim-3 could mediate CD4+ cell depletion [17] which promoted the growth of tumor cells [18, 24]. Accumulating evidence has supported that Tim-3 expression is a marker for the most dysfunctional T cell population in humans [43] and also in a mouse model of HBV infection [42]. It can also serve as a prognostic marker for solid tumours, where survival is negatively correlated with the presence of Tim 3+ T cells [12]. Human Tim-3 is a transmembrane protein in membrane or a soluble form, which can be cleaved from the cell surface by some matrix metalloproteinases to a soluble form [44], but the exact function of sTim-3 is not well defined. Elevated plasma levels of sTim-3 appear to correlate with enhanced viral load in patients with HIV infection [8]. According to the previous description, sTim-3 may serve as a biomarker for patients with osteosarcoma [45] and graft-versus-host disease [46]. Increased serum sTim-3 was related to the progression of hepatitis virus infection diseases [47], which indicated that Tim-3 can act as a biomarker beside as a clinical target. On this basis, Tim-3 was considered to be closely associated with the clinical prognosis of chronic HBV infection [48], which could be used as a new index to evaluate the prognosis in CHB patients.

Our data showed that serum Tim-3 was highly expressed in CHB patients, which presented reduction after continuous antiviral treatment. Serum Tim-3 was positively correlated with serum ALT, AST, TB, DB, TBA, γ-GT, AFP, rather than HBV-DNA. There was a close relationship between Tim-3 expression and the degree of liver inflammation. Combined with the pathological results, serum Tim-3 level was closely related to liver histological activity. Under some specific conditions, such as normal liver function or negative HBeAg in chronic hepatitis B patients, Tim-3 was more suitable to reflect the pathological changes in liver tissues compared with ALT and AST. Tim-3 expression was closely related to the changes of liver pathological inflammation grade and fibrosis degree. Their alternation trends were similar. However, much remains to be learned about the specific mechanisms in it, particularly in infectious diseases. Nevertheless, the changes of Tim-3 promoted the evaluation of the disease and the judgment of prognosis for CHB patients with normal liver function.In future, Tim-3 may serve as a candidate for treating CHB and a prognostic serum molecular marker, which is crucial for the development of individualized treatment plans. Our study provides some new insights into the establishing of Tim-3 based regimens for treating chronic HBV infection.

## Data Availability

All data generated or analysed during this study are included in this published article.

## Abbreviations

AFP: Alpha-fetoprotein
ALB: Albumin
ALD: Alcohol-related liver disease
ALP: Alkaline phosphatase
ALT: Alanine aminotransferase
anti-HBc: Antibodies against hepatitis B core antigen
anti-Hbe: Antibodies against hepatitis B e antigen
anti-HBs: Antibodies against hepatitis B surface antigen
APCs: Antigen presenting cells
AST: Aminotransferase
CEACAM1: Carcinoembryonic antigen cell adhesion molecule 1
CHB: Chronic hepatitis B
ChE: Cholinesterase
CR: Checkpoint receptor
DBIL: Direct bilirubin
Gal-9: Galectin-9
GLB: Globulin
GPDA: Glycyl-proline-dipeptidyl aminopeptidase
HbeAg: Hepatitis B e antigen
HbsAg: Hepatitis B surface antigen
HBV: Hepatitis B virus
HBV-DNA: Hepatitis B virus-deoxyribonucleic acid
HCC: Hepatocellular carcinoma
HMGB1: High mobility group protein B1
IDBIL: Indirect bilirubin
IFN-γ: Interferon-gamma
IgV: Immunoglobulin variable domain
KCs: Kupffer cells
LC: Liver cirrhosis
LF: Liver failure
PtdSer: Phosphatidylserine
RICD: Restimulation-induced cell death
sTim-3: Soluble Tim-3
TBA: Total bile acids
TBIL: Total bilirubin
Th1: Type I helper T lymphocytes
Tim-3: T-cell immunoglobulin and mucin domain containing molecule 3
TP: Total protein
γ-GT: Glutamyl transpeptidase
